# How to Make a Fast, Efficient Bubble-Driven Micromotor: A Mechanical View

**DOI:** 10.3390/mi8090267

**Published:** 2017-08-30

**Authors:** Lisheng Liu, Tao Bai, Qingjia Chi, Zhen Wang, Shuang Xu, Qiwen Liu, Qiang Wang

**Affiliations:** 1 State Key Laboratory of Advanced Technology for Materials Synthesis and Processing, Wuhan University of Technology, Wuhan 430070, China; 2 Department of Mechanics and Engineering Structure, Wuhan University of Technology, Wuhan 430070, China; whut_baitao@163.com (T.B.); qingjia@whut.edu.cn (Q.C.); wangzhen@whut.edu.cn (Z.W.); xu_shuang@whut.edu.cn (S.X.); qiwen_liu@whut.edu.cn (Q.L.); 3 Infrastructure Management Department, Wuhan University of Technology, Wuhan 430070, China; qiang_wang@whut.edu.cn

**Keywords:** bubble-driven micromotors, dynamic mechanism, geometric design, environmental factor

## Abstract

Micromotors, which can be moved at a micron scale, have special functions and can perform microscopic tasks. They have a wide range of applications in various fields with the advantages of small size and high efficiency. Both high speed and efficiency for micromotors are required in various conditions. However, the dynamical mechanism of bubble-driven micromotors movement is not clear, owing to various factors affecting the movement of micromotors. This paper reviews various factors acting on micromotor movement, and summarizes appropriate methods to improve the velocity and efficiency of bubble-driven micromotors, from a mechanical view. The dynamical factors that have significant influence on the hydrodynamic performance of micromotors could be divided into two categories: environment and geometry. Improving environment temperature and decreasing viscosity of fluid accelerate the velocity of motors. Under certain conditions, raising the concentration of hydrogen peroxide is applied. However, a high concentration of hydrogen peroxide is not applicable. In the environment of low concentration, changing the geometry of micromotors is an effective mean to improve the velocity of micromotors. Increasing semi-cone angle and reducing the ratio of length to radius for tubular and rod micromotors are propitious to increase the speed of micromotors. For Janus micromotors, reducing the mass by changing the shape into capsule and shell, and increasing the surface roughness, is applied. This review could provide references for improving the velocity and efficiency of micromotors.

## 1. Introduction

Inspired by nature and dependent on the development of nanotechnology, micromotors have been in development for decades. Efficient micromotors offer great potential in biochemical and biomedical applications. Therefore, lots of researchers have strived hard to study the dynamic behaviors and improve the efficiency of different kinds of micromotors to meet various requirements. The development of micromotors is a significant advancement towards the realization of micro/nanoscale world. In 2002, Whitesides et al. [1] created self-propelling plates, which are the prototype of micromotors and the beginning of the development of micromotors. Micromotors are microscale structures which convert different sources of energy into kinetic energy, and perform tasks in a micro/nanoscale world. They have been widely used in environmental chemistry [2,3], drug delivery [4,5,6,7], and cell separation [8,9,10]. Over the past years, researchers have invented several kinds of bubble-driven micro/nanomotors. There are three propulsion models of bubble-driven micromotors, according to the driving mechanisms: self-electrophoresis [11,12,13,14], self-diffusiophoresis [15,16,17,18], and bubble propulsion [19,20]. Most bubble-driven micromotors convert chemical energy into kinetic energy, utilizing bubbles to drive the micromotors. A recoil could be produced at the end of micromotors, caused by the generation and growth of bubbles. Bubbles are the medium in the conversion of chemical energy into kinetic energy.

**Figure 1:**
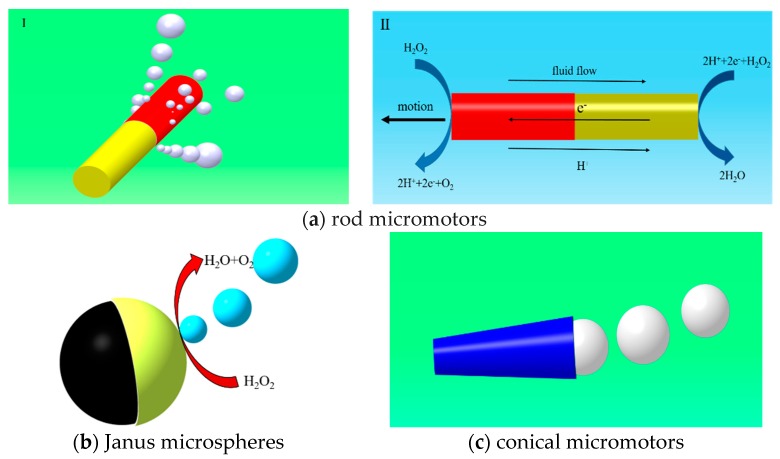
The proposed mechanisms for micromotors of different geometries. (**a**) There are two propelled mechanisms for rod micromotors: (I) the rod micromotors are propelled by bubbles generated at the surface at one end of microrods [23], and (II) the electrokinetic mechanisms of microrods [24]; (**b**) bimetal Janus microspheres propelled by diffusing of bubbles at the surface of microspheres; (**c**) conical micromotors propelled by growth and jet of bubbles generated by chemical catalytic reactions.

In all kinds of micromotors, bubble-driven micromotors are mostly notable for their small size, light-weight, high thrust–weight ratio, and low energy consumption. Most bubble-driven micromotors are fabricated by a rolled-up technique [19,20] or template electro synthesis [21,22], with advantages of high speed and propulsion force. The speed of these micromotors could reach thousands of micrometers per second, much faster than other kinds of micromotors. The propelling of bubble-driven micromotors decides the motion and the dynamic behavior of micromotors. For self-electrophoresis micromotors, such as bimetallic micromotors, two different electrochemical reactions, which proceed with electron flows, result in propelling the moving micromotors, as shown in Figure 1a. As for self-diffusiophoretic micromotors, the existence of concentration gradients around the micromotors drive the micromotors forward, as shown in Figure 1b. For most of self-diffusiophoresis and self-diffusiophoretic micromotors, bubbles are mainly involved in pronounced concentration gradients, and continuously dissolve into the fluid. Therefore, bubbles are usually not unambiguously identified as discrete propulsion units. In Figure 1c, bubble-propelled micromotors are driven by the bubble generation and injection, which is caused by a catalytic reaction with the chemical fuels. This mechanism leads to the unique stop-and-go propulsion behavior of bubble-propelled micromotors observed at low Reynolds numbers [19].

Given certain solution environments, optimizing the geometry of motors is available to improve the velocity of motors. Up to now, bubble-driven micromotors of different geometries are varied, including conical micromotors [25], Janus microspheres [26,27], rod micromotors [28,29], and motors with other geometries [30]. The geometry of micromotors plays an important role on the velocity and movement of micromotors [31]. At the surface of Janus and rod micromotors, the bubbles generate and diffuse into the fuel, due to the concentration gradient of bubbles. Most of the Janus and rod micromotors are propelled when bubbles are released into the fuel. In order to enhance the bubble nucleation, shell micromotors [32] and conical micromotors [19,20] are created, which have catalyst coated inside the shell or inner wall of microjets. Different from solid micromotors, shell micromotors and conical micromotors have concave cavities where the bubbles generate and grow. When the radius of bubbles is large enough, the bubbles separate and jet from the end of micromotors, where bubbles are easily disengaged. The micromotor is driven by the generation and injection of bubbles.

Currently the propellant mechanism of bubble-driven micromotors is unclear. Related research was typically carried out from the views of material and chemistry. Researchers prefer to use the method of increasing the concentration of hydrogen peroxide to improve the velocity of bubble-driven micromotors, but high concentrations of hydrogen peroxide are not achievable, due to strong oxidation. Spherical and rod are not the best shapes for micromotors, with a large resistance coefficient, in the view of hydrodynamics. Therefore, reducing the resistance coefficient of bubble-driven micromotors by optimizing the geometry of motors, is an effective method to improve the mechanical behavior and efficiency of bubble-driven micromotors.

Meanwhile, the movement of bubble-driven micromotors is a complex fluid–solid coupling process. The flow field around the micromotors could also affect the dynamic behavior of bubble-driven micromotors. Due to the complexity of surrounding flow field, it is difficult to propel microscale objects at the existence of low Reynolds number, viscous forces, and Brownian motion in liquid [33]. The relationship among various factors, which affect the dynamic behavior of micromotors, is complex. Therefore, the dynamic behaviors of different kinds of micromotors and the factors influencing the behavior of micromotors are concluded in this paper. These factors are divided into two categories. One is the environmental parameters, including concentration and viscosity, and the other one is the geometrical factors, including semi-cone angle and the ratio of length to larger radius of tubular micromotors. This paper can provide references for improving the velocity and efficiency of micromotors.

## 2. Environmental Factors

Various fuels, including hydrogen peroxide, water [34], acids [35,36] and hydrazine [37,38], are applied to drive micromotors. Besides, there are several micromotors propelled by external energy, including light energy [39,40,41], magnetic energy [42,43,44,45,46] and other propellant forms [47,48]. Therefore, the change of solution, including temperature, viscosity and concentration of solution, could affect the mechanical behaviors of motors. Different micromotors demonstrate different dynamic behaviors according to the environment they localize [49,50].

### 2.1. Concentration and Type of Fuel

**Figure 2:**
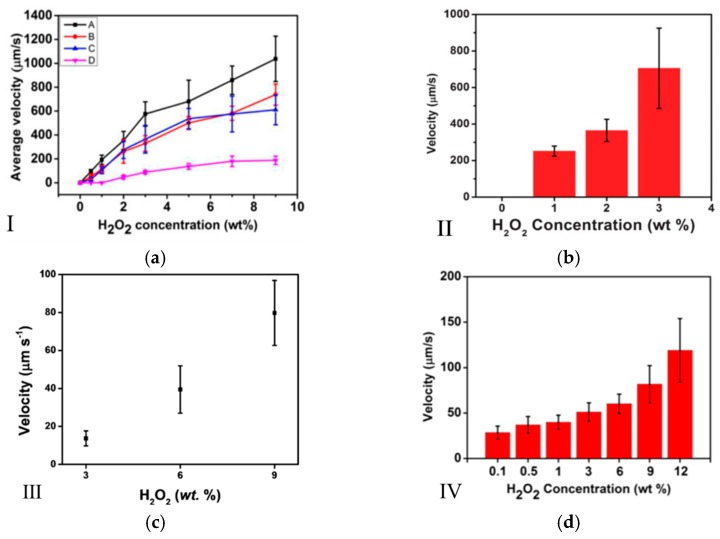
The relationship between the concentration of H_2_O_2_ and micromotors of different shapes. (**a**) The dependence of the average velocity of the four type of tubular micromotors on the concentration of H_2_O_2_ [59]. (A. Electrodeposited in 5 μm pores; B. Electrodeposited in 2 μm pores; C. Electrodeposited in 2 μm pores (shorter deposition time); D. Electrodeposited in 200 nm pores.) (**b**) The relationship between the average velocities of microjets and the concentration of fuel [53]. (**c**) The dependence of the velocity of Janus Ag micromotors on the hydrogen peroxide concentration [60]. (**d**) The relationship between the average velocity of Ag catalytic micromotors on H_2_O_2_ concentration at 23 °C [61]. Reproduced with permission from [53,59,60,61].

Bubble-driven micromotors move in hydrogen peroxide solution in the action of chemical reactions. They are propelled by bubbles in different concentrations of hydrogen peroxide fuel. A high concentration of hydrogen peroxide promotes high-speed movement of micromotors. A kind of catalytic microtubular using bilayer polyaniline (PANI)/Pt was fabricated with the highest speed of 3 mm/s [22]. Moreover, a relationship between the speed of highly efficient catalytic microtubular engines, and the concentration of hydrogen peroxide fuel, has been proposed. It indicated that the main factors affecting the speed of micromotors are radius and frequency of the bubble, and both of them are influenced by the concentration of peroxide fuel. With the hydrogen peroxide level rising from 0.3% to 0.5%, the bubble frequency increased, while the bubble size decreased. The speed of micromotor decreased due to larger bubbles with lower frequency. It was also found that the speed of micromotors is related to the surface tension, bubble size, and bubble frequency influenced by the concentration of the solution. For Janus micromotors, SiO_2_@rGO-Pt Janus micromotors move faster in the solution as the concentration of hydrogen peroxide increases [51]. The maximum speed can reach 725 ± 42 μm/s at 10% concentration of H_2_O_2_ fuel. At different concentrations of H_2_O_2_, the increased velocity on the increasing concentration of hydrogen peroxide was independent of the geometry of micromotors. Besides, the rise of velocity slowed down as H_2_O_2_ concentration increased, as shown in Figure 2a. Scholars have used acid solutions instead of hydrogen peroxide. Wang et al. created a new kind of micromotor, Zn-based microrockets [35]. In order to prolong the life of micromotors, poly (3,4-ethylenedioxythiophene) (PEDOT)/Zn bilayer structure was applied on the Zn-based microrockets [52]. The PEDOT/Zn micromotors can be self-driven for ~10 min in a simulated gastric acid (pH up to 2).

However, high concentrations of hydrogen peroxide and acid solutions are not applicable because of their strong oxidation. More and more researchers investigated more practical self-propelled micromotors in biological systems. The Cu/Pt concentric bimetallic microjet engines show fast moving speeds of ~7 body length per second in low concentrations of hydrogen peroxide (0.2%) [53]. Due to the strong oxidation and corrosion effects of most fuels, clean fuel is urgently required. To reach the goal of generating bubbles providing driving power, the reaction between water and active metal is studied in recent years. Water is the most potentially clean fuel, a status which is easily achieved as it is the necessary liquid for life. A new kind of bubble-propelled micromotor driven by water was created [54]. The micromotor was constituted of Al–Ga binary alloy microsphere utilizing aluminum microparticles and liquid gallium mixing. They are propelled by bubbles which are generated by the reactions between aluminum and water. The first example of water-driven micromotor could reach 3 mm/s. Afterwards, another water-driven micromotor propelled by Mg–water reactions was created. A Mg/Pt Janus micromotor, with an average speed of 75.7 μm/s, was designed [55]. Based on a redox reaction of magnesium with water, TiO_2_/Au/Mg microspheres were invented [56]. The water-driven TiO_2_/Au/Mg micromotor could move at the speed of 110 μm/s, with a size of 20 μm. To enhance the ability of removing a wide range of contaminants from polluted water, new kinds of micromotors were created [57]. On the basis of the ability of activated carbon microparticles to remove toxic heavy metals, self-propelled activated carbon Janus micromotors were created. The activated carbon Janus micromotors could move with a speed more than 500 μm/s. Considering the biomedical environment of the micromotors, different concentrations of bovine serum albumin (BSA) were applied to the PEDOT/MnO_2_ micromotor [58]. In order to improve the application ability of micromotors, research on the material and fuel is developing.

### 2.2. Viscosity

The environments with implications for the micromotors are different. In practical media, which often includes blood or oil, the viscosity is largely different from that of water. Besides, the viscous effect of the fluid is obvious, due to low Reynolds number in the movement of the micromotor. Consequently, with the viscosity of solution increasing, the drag force of micromotors increases, and finally, the velocity decreases.

**Figure 3:**
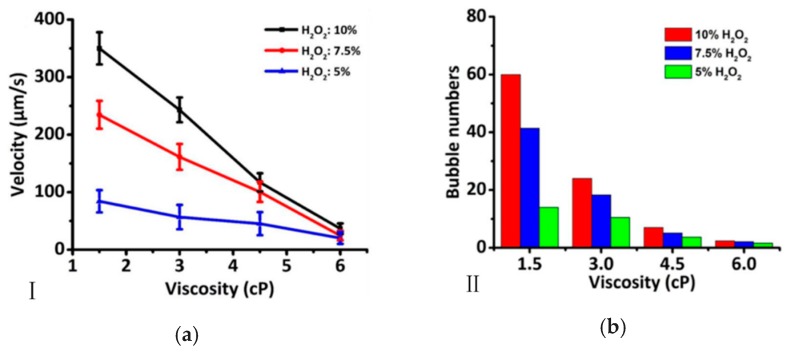
The influence of solution viscosity on the dynamic behavior of micromotors. (**a**) The velocity of micromotors decreases with the increase of solution viscosity; (**b**) the relationship between the frequency of bubble injection and the viscosity of fuel [62]. Reproduced with permission from [62].

A linear relationship between the velocity of microrocket and the viscosity of solution was revealed by experimental and theoretical methods [62]. Meanwhile, the bubble diameter was larger and bubble numbers decreased as the viscosity increased. Another set of experimental data indicated that an increasing biological media viscosity causes the speed of the micromotors to decrease [58]. Also, a relationship between Reynolds number and the dynamic behavior was built by Pumera [63]. The Reynolds number strongly depends on the viscosity of the solution and velocity of the micromotor. The motion of the micromotors was linear at a higher viscosity with lower Reynolds, while the motion was circular at a lower viscosity with larger Reynolds numbers. Figure 3 shows that the motion of micromotors was strongly depended on viscosity and Reynolds number. From the theory of fluid mechanics by Stokes’ drag theory, it is obtained that the drag force of the micromotor is stronger as the viscosity of the solution increasing. Therefore, the velocity of the micromotor decreases with increasing viscosity.

In some studies, the velocity of micromotors was independent of the solution viscosity [64,65]. Some hydrodynamic models of self-propelled micromotors, describing bubble growth, were proposed from theoretical calculation and experiment results. The surface tension force, inertial force and gas momentum force were neglected, which caused viscosity term eliminated. Notably, in the motions of low Reynolds number, the surface tension force cannot be ignored under mico–nano dimensions.

Further studies on propulsion mechanisms of bubble-driven micromotor for motion control have been inspired. A polymer-based chemical locomotive using H_2_O_2_ as chemical fuel has been created [66]. The relationship between the vertical velocity of the locomotive and the viscosity of fuels was established. Furthermore, the vertical velocity could be controlled by changing the viscosity according to this proportional character of the velocity of the locomotive and the viscosity of medium.

### 2.3. Temperature

As one of the main environmental factors, temperature is rarely studied by researchers. Most chemical reactions are affected by temperature. The velocity of micromotors rises with increasing temperature. Sanchez suggested that temperature could be employed to control the efficiency of micromotors [67]. With the temperature changing, the dynamical behavior was modeled. The speed of micromotors increased between 5 and 20 °C. When the temperature is higher than 20 °C, the relationship between temperature and speed follows a linear trend. Simultaneously, the dynamic viscosity of solution is reduced, from 1.7 to 0.9 mPa·S when the temperature increases, from 5 to 37 °C. As the viscosity decreases, the speed of micromotors increased. From the experiment, it was found that the motion of the micromotors was linear at lower temperature, with higher viscosity, while the motion was circular at higher temperature, with lower viscosity. An empirical model of the temperature-dependent dynamics of micromotors was built to describe the dynamic behavior of micromotors propelled by bubbles. Thus, the temperature of the solution could be controlled to improve the efficiency of the micromotors, and keep more cell-friendly environmental self-propelled micromotors at high speeds.

## 3. Geometric Design of Micromotor

After optimizing environmental factors and introducing clean solutions, geometry of design should be introduced to improve the velocity of micromotors. Bubble-driven micromotors of different geometries were manufactured [68,69,70,71]. As Table 1 shown, Se et al. created bimetallic nanorods propelled by catalytic decomposition of hydrogen peroxide [72,73]. Wang [74] and Pumera [75] improve rod motors into tubular microengines. Furthermore, the geometry of micromotors is constantly improved to enhance the dynamic performance of micromotors. Conical motors are developed and applied [76,77]. Meanwhile, the different dynamic behaviors of Janus microspheres [78,79,80] and motors with other shapes [81,82,83,84] are studied. According to the theory of Mitrovic, the asymmetry of bubbles causes a pressure difference (Laplace pressure) [85] across the bubble interface, which drives the bubbles moving along the inner wall. The geometry of the micromotor can affect the flow field and pressure distribution when the micromotor is propelled. The speed of the micromotor is determined by the balance between drag forces and driving forces acting on the micromotor. At the accelerating stage of the micromotor, the velocity of micromotor increases, with the drag force increasing. When the drag force and the driving force acting on the micromotor reach the equilibrium point, the micromotor will be at its highest speed. Therefore, rational design of the geometry can increase the efficiency and the velocity of the micromotor, which requires theoretical methods of fundamental mechanisms and fluid mechanics.

### 3.1. Tubular and Rod Micromotors

#### 3.1.1. Semi-Cone Angle

Semi-cone angle is typical of tubular micromotors, which affects the drag coefficient. Based on the experiments, the drag coefficient decreases as the semi-cone angle increases, which causes the velocity of micromotor to increase. A conical shaped micromotor could get a higher speed than a cylindrical micromotor with the same length and radius [86]. The shape factor, which is related to the surface’s geometry and chemical properties, of concave surfaces, is smaller than that of convex surfaces [32]. In the work Wang et al., the velocity of micromotors increased with the increase of the semi-cone angle [87]. The semi-cone angle affects the shape factor in concave surfaces and convex surfaces. It is easier for bubbles to detach from the micromotor in depending on different shape factors, where they more readily detach from concave compared to convex surfaces. According to the theory, the semi-cone angle can affect the shape factor to change the bubble size and generation frequency. Besides, the large semi-cone angle of micromotor can enlarge the contact area of micromotors to get more chemical energy from the catalytic reaction.

In another study, the average velocity of a micromotor is strongly dependent on the semi-cone angle, which has a significant effect on the expelling frequency for conical tubular micromotors [64]. However, the conclusion of velocity is the opposite. The average velocity of a micromotor was the result of step displacement and the bubble ejection frequency. The microjet velocity and the drag force acting on the micromotor decreased as the semi-cone angle of the micromotor increased from 0° to 5°.

It is worth nothing that the semi-cone angle is closely related to the motion of micromotors. Reasonable design of the semi-cone angle of microjet by hydrodynamics could improve the efficiency of bubble-driven microjets and utilization of fuel.

#### 3.1.2. The Ratio of Length to Larger Radius

Another geometric factor of tubular micromotors is the ratio of length to larger radius (ξ = L/Rmax). However, the relationship between the ratio ξ and the velocity of micromotors possesses singularity. A recent theory identified that the speed of the micromotor is almost equal to the product of the bubble radius and frequency [20]. Therefore, rational design of the ratio, to increase the bubble radius and frequency, is necessary.

According to experiment data of micromotors with different ratio, Li [86] proposed a model describing the relationship between the ratio, ξ, and the drag coefficient. The drag coefficient decreased as the ratio ξ increased, while less than 3, and it increased as the ratio increased to greater than 6. According to the shape equation, the ratio of length to radius could seriously affect the dynamic shape and structure of the micromotor. In order to get higher speeds, a conical shaped micromotor is the best choice, instead of any other shape of micromotor. Decreasing the ratio of length to larger radius could reduce the drag force acting on the micromotor and improve the velocity. The motion of the micromotors with solid structures is dependent on the height to width ratio [92]. In the experiment, the motion of the micromotor tended to be more spiral, as the height to width ratio increased with the same height of micromotor. Besides, in another theory, the speed of the micromotor depended closely on the dimensions and the radius of the micromotor [93]. In their experiments, the frequency for bubble ejection and the maximum O_2_ concentration increased as the length of micromotor increased. Therefore, the velocity of micromotor increases with increasing length. A model about addressing how the geometric dimensions of the micromotor affect the dynamic characteristics was built. The velocity of the microjet decreased with either increasing radius or decreasing length [94]. The surface area increased with the length of the micromotors, resulting in higher oxygen production and improved velocity of the micromotors. However, as the velocity increased, the drag force acting on the micromotor also increased, which slowed down the velocity of the micromotor. According to the experiment data [94], the velocity of micromotor increased with the length of the micromotors, when less than 200 μm, and there was a dramatic velocity drop in experimental results for lengths of micromotors greater than 200 μm. For the microjets with a small radius, the velocity of micromotors decreased with the increasing length of micromotors. However, for the micromotors with large radii, the velocity of micromotors reduced at the small lengths, and the maximum value of velocity was achieved at lengths from 40 to 200 μm.

Various length to larger radius ratios of tubular micromotors have been examined. The relationship between the velocity and the geometries of tubular and rod micromotors is complex. Further study on the dependence on both is required.

### 3.2. Janus Micromotors

Janus micromotors have several structures, such as bimetallic structures, shell, and capsule micromotors. A bimetallic Janus micromotor is driven by the catalytic reaction between the surface and the solution surrounding the micromotor. The speed of self-propulsion micromotors was 60 μm/s and more [95]. The Janus micromotor was recognized as being composed of two hemispherical domains: passive part and reactive part. Janus micromotors moved forward due to the bubble growth and separation, which were produced in the reactive part. The micromotors moved forward due to the growth force when the bubble was growing, while the micromotor was pulled backward as a result of an instantaneous local pressure depression when the bubble burst [96]. A model based on oxygen bubble detachment has been built [97]. According to the model, the velocity of Janus micromotor increased along with increasing surface tension and concentration of hydrogen peroxide.

However, the speed of Janus micromotors is improving easily by changing their shape. Changing the shape of Janus micromotors into multilayer hollow capsules, which were fabricated by a template-assisted layer-by-layer self-assembly [89], created a new kind of self-propelled micromotor. The Janus capsule motor they created could move over 125 body lengths/s at speeds of above 1 mm/s. Combining with biomolecular proteins, a new kind of Janus micromotor was fabricated, which is composed of a chitosan and alginate multilayer capsule [98]. The capsule micromotors were produced by conventional template-assisted layer-by-layer self-assembly. The micromotor could achieve a speed of 23.27 μm/s, with a diameter of 5 μm. In order to reduce the mass and form a cavity, the nanoshell micromotor is fabricated [32] Depending on the speed of 100 μm/s, the nanoshell micromotor was superior to solid, spherical Janus motors. By developing the nanoshell micromotor, a catalytic polymer multilayer shell motor was manufactured. The new multilayer shell motor utilized layer-by-layer self-assembly to sputter platinum onto the surface of silica templates [99]. It could at the speed of 260 μm/s, 13 body lengths/s. More studies reported that roughening the surface of the Janus micromotors could improve the reaction and make the micromotor move faster. Enzyme-powered hollow mesoporous Janus nanomotors, which were covered in different enzymes on the surface, were mentioned [100]. Three different enzymes were tested, and the apparent diffusion coefficient was obviously enhanced. Based on the same principles, graphene-wrapped micromotors were fabricated, which conducted the surface by introducing an extra layer of reduced graphene oxide (RGO) [101]. The graphene-wrapped micromotors could move faster than the common Au–Pt Janus motors in the same concentration of H_2_O_2_, due to the increase in the conduciveness of surface. By utilizing the cationic surfactant to form the hollow mesoporous structure, a hollow mesoporous silica sphere was created [102]. The cationic surfactant assisted silica spheres had large a surface area, high pore volume, and controllable structure parameters, which could made the micromotors move faster and more efficiently.

### 3.3. Surface Roughness

Lots of Janus micromotors move faster due to their inherent surface roughness, which resulted in a microporous catalyst patch with an increased area for enhanced fuel decomposition. In earlier work, Merkoci et al. discovered that the surface of SiO_2_@rGO–Pt Janus micromotors showed corrugated and scrolled nanosheets that resembled crumples [51]. Therefore, increasing surface roughness became a means of promoting the speed of Janus micromotors. A new bubble-propelled activated carbon Janus micromotor was created by Wang et al. [57] The rough surface of the activated carbon microsphere forms a microporous Pt structure, which provides a highly catalytic layer. The tiny change in the structure leads to effective bubble evolution and propulsion at the speed of over 500 μm/s. Inspired by increasing surface roughness of Janus micromotors, the method is also applied to tubular micromotors. However, the speed of carbon-based micromotors is a compromise between two opposite forces: the increased driving force by improving fuel decomposition in the inner catalytic layer and the friction force at the rough outer surface with the fluid [21]. Increasing the surface roughness of C_60_ fullerene and carbon black–Pt micromotors leads to a large friction force at the outer surface, which results in a reduced speed of ~180 μm/s in 1% hydrogen peroxide solution. In contrast, the catalytic driving force is the dominant force for carbon–nanotube–Pt micromotors, which cause the ultrafast speeds up to 440 μm/s in 1% hydrogen peroxide solution. Wang et al. applied CdS and ZnS nanocrystals on tubular micromotors, which resulted in a rough Pt catalytic surface [103]. The speed of CdS–polyaniline–Pt and ZnS–polyaniline–Pt micromotors could be raised to 2500 μm/s, due to highly efficient bubble propulsion.

Accordingly, for Janus micromotors, increasing surface roughness is an effective means to increase the speed of the Janus micromotors. However, for tubular micromotors, the friction force at the rough outer surface with the fluid may become the dominant force in the movement. In the design and fabrication of tubular micromotors, we should avoid increased surface roughness at outer surfaces when improving the roughness at the inner surface of tubular micromotors. Based on this design method, the speed of tubular micromotors should be improved for highly efficient catalytic reactions at inner surfaces, with a small friction force at outer surfaces of tubular micromotors.

## 4. Conclusions and Perspectives

**Figure 4:**
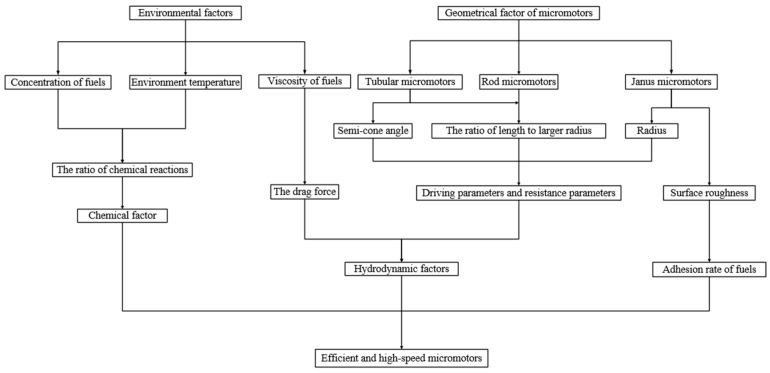
Different factors affect the dynamic behavior and the efficiency of bubble-driven micromotors. The factors influencing the dynamic behavior of micromotors are divided into environmental factors and geometrical factors of micromotors. From another viewpoint, the factors could also be divided into chemical factors and hydrodynamic factors. The hydrodynamic factors consist of the size of micromotors and the viscosity of solution, and chemical factors mainly include the concentration of fuels and the environment temperature.

The motion of micromotors is a complex fluid–solid coupling process. Therefore, it is necessary to consider the problem considering both the micromotor and the flow fluid. Different geometries of micromotors and flow environments around micromotors have been reviewed from the view of mechanics. Figure 4 summarizes all the hydrodynamic and chemical factors influencing the dynamic behavior of bubble-driven micromotors.

Firstly, increasing the concentration of hydrogen peroxide and environment temperature, or decreasing the viscosity, can be used to improve the velocity of micromotors. However, high concentrations of hydrogen peroxide are not practical for living organisms. For this purpose, nontoxic and clean fuels should be applied, meanwhile, new materials for the micromotor should be developed. In the environment of low concentration, geometry of micromotors should be concerned with improving the velocity of motors. For tubular and rod micromotors, it is certain that the speed of tubular micromotors is faster than that of rod solid micromotors. Meanwhile, with larger semi-cone angle and smaller ratios of length to radius, the tubular micromotor could achieve higher speeds. In Janus micromotors, changing their shape is more effective to improve efficiency. Shell and capsule are the best choices for Janus micromotors, with the effect of reducing mass. Besides, it is doubtless that increasing the surface roughness of micromotors is also an effective way to improve the speed of micromotors. For Janus micromotors, increasing surface roughness is an effective means to increase the speed of the Janus micromotors. However, for tubular micromotors, we should avoid the increase in surface roughness at the outer surface when improving the roughness at inner surface. In this way, the speed of tubular micromotors should be improved for highly efficient catalytic reactions at the inner surface, and with small friction force at the outer surface of tubular micromotors.

Current methods rely on the experiments, and lack accurate theoretical analysis or methods which describe the dynamic mechanism of bubble-driven micromotors. The relationship among fluid, micromotors, and bubbles is ignored in current studies. The motion of micromotors propelled by the growth and jet of bubbles is the fluid–solid coupling problem with low Reynolds number. The influence of fluid should be studied and introduced into the research of micromotor movement. Meanwhile, in the current studies of micromotors, irregular Brownian movement is neglected. Due to the small size of micromotors, dimensional effects could influence the movement of micromotors. We hope that this mechanism provides a reference for the practical application of micromotors and a basis for the dynamic theory of micromotors.

## Figures and Tables

**Table 1:** Speed of bubble-driven microomtors. Reproduced with permission from [32,52,54,58,59,64,75,88,89,90,91].

Type of the Micromotors	Morphology	Condition	Schematic	Length	Radius	Max. Speed
Janus micromotors [88]	Al/Pd bimetallic Janus micromotors	Strong acid (HCl, 100 mM) and strong base (NaOH, 100 mM)	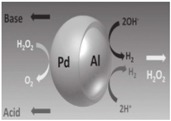		2.5–15 μm	200 μm/s
Tubular micomotors [52]	PEDOT/Zn tubular micromotors	Under the gastric acidic condition (pH up to 2) at phsiological temperature	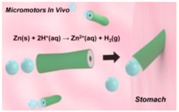	20 μm	5 μm	60 μm/s
Conical micromotors [58]	PEDOT/MnO_2_ tubular micromotors	In very low levels of hydrogen peroxide down to 0.4%	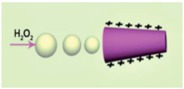	8 μm	1 μm	318.8 μm/s
Tubular micromotors [59]	Pure platinum micro/nanotubes	At 1–3% concentration of hydrogen peroxide.	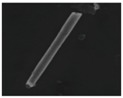	15.68 μm	4.66 μm	379.77 μm/s
Tubular microengines [75]	Tubular microengines using fruit tissue cells as the support of the deposited metal layer	In 3% concentration of hydrogen peroxide	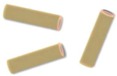	50 μm	1.5 μm	1000 μm/s
Shell micromotors [32]	Au/Ag/Pt nanoshell micromotors	In 5% concentration of hydrogen peroxide	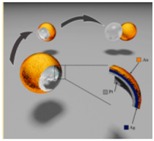		1–15 μm	100 μm/s
Conical micromotors [64]	PEDOT/platinum multilayer conical micromotors	In 1% concentration of hydrogen peroxide containing 1% Triton X-100	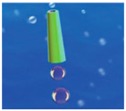	100 μm	10 μm	100 μm/s
Janus micromotots [54]	Al–Ga/Ti Janus microparticles	Pure water	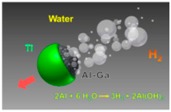		10 μm	3000 μm/s
Janus capsule micromotors [89]	Partially coated dendritic platinum nanoparticles	At 30% concentration of hydrogen peroxide	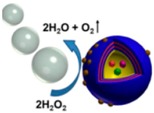		8 μm	1000 μm/s
Rod micromotors [90]	Catalytic Pt–Au nanorod motors	At 3% concentration of hydrogen peroxide	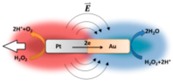	100 μm	1 μm	50 μm/s
Carbon nanotube [91]	Au/Pt carbon nanotubes	At 15 wt % aqueous H_2_O_2_ fuel	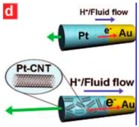	1 μm	0.11 μm	50–60 μm/s (up to above 200 μm/s)
